# A review of the explainability and safety of conversational agents for mental health to identify avenues for improvement

**DOI:** 10.3389/frai.2023.1229805

**Published:** 2023-10-12

**Authors:** Surjodeep Sarkar, Manas Gaur, Lujie Karen Chen, Muskan Garg, Biplav Srivastava

**Affiliations:** ^1^Department of Computer Science and Electrical Engineering, University of Maryland, Baltimore County, Baltimore, MD, United States; ^2^Department of Information Systems, University of Maryland, Baltimore County, Baltimore, MD, United States; ^3^Department of AI & Informatics, Mayo Clinic, Rochester, MN, United States; ^4^AI Institute, University of South Carolina, Columbia, SC, United States

**Keywords:** explainable AI, safety, conversational AI, evaluation metrics, knowledge-infused learning, mental health

## Abstract

Virtual Mental Health Assistants (VMHAs) continuously evolve to support the overloaded global healthcare system, which receives approximately 60 million primary care visits and 6 million emergency room visits annually. These systems, developed by clinical psychologists, psychiatrists, and AI researchers, are designed to aid in Cognitive Behavioral Therapy (CBT). The main focus of VMHAs is to provide relevant information to mental health professionals (MHPs) and engage in meaningful conversations to support individuals with mental health conditions. However, certain gaps prevent VMHAs from fully delivering on their promise during active communications. One of the gaps is their inability to explain their decisions to patients and MHPs, making conversations less trustworthy. Additionally, VMHAs can be vulnerable in providing unsafe responses to patient queries, further undermining their reliability. In this review, we assess the current state of VMHAs on the grounds of user-level explainability and safety, a set of desired properties for the broader adoption of VMHAs. This includes the examination of ChatGPT, a conversation agent developed on AI-driven models: GPT3.5 and GPT-4, that has been proposed for use in providing mental health services. By harnessing the collaborative and impactful contributions of AI, natural language processing, and the mental health professionals (MHPs) community, the review identifies opportunities for technological progress in VMHAs to ensure their capabilities include explainable and safe behaviors. It also emphasizes the importance of measures to guarantee that these advancements align with the promise of fostering trustworthy conversations.

## 1. Introduction

Mental illness is a global concern, constituting a significant cause of distress in people's lives and impacting society's health and well-being, thereby projecting serious challenges for mental health professionals (MHPs) (Zhang et al., 2022). According to the National Survey on Drug Use and Health, nearly one in five US adults lives with a mental illness (52.9 million in 2020) (SAMHSA, 2020). The reports released in August 2021 indicate that *1.6 million people* in England were on waiting lists to seek professional help with mental healthcare (Campbell, 2021). The disproportionate increase in the number of patients in comparison to MHPs made it necessary to employ various methods for informative healthcare. These methods included (a) public health forums such as Dialogue4Health, (b) online communities such as the r/depression subreddit on Reddit, (c) Talklife (Kruzan, 2019), and (d) Virtual Mental Health Assistants (VMHAs) (Fitzpatrick et al., 2017). By operating anonymously, these platforms (a, b, c) effectively eliminated the psychological stigma associated with seeking help, which had previously deterred patients from consulting an MHP (Hyman, 2008). Furthermore, the absence of alternative sources for interpersonal interactions led to the necessity of developing Virtual Mental Health Assistants (VMHAs) (Seitz et al., 2022).

**VMHAs**: Virtual Mental Health Assistants (VMHAs) are AI-based agents designed to provide emotional support and assist in mental health-related conversations. Their primary objective is to engage in organized conversation flows to assess users' mental health issues and gather details about the causes, symptoms, treatment options, and relevant medications. The information collected is subsequently shared with MHPs, to provide insights into the user's condition (Hartmann et al., 2019). VMHAs are a valuable and distinct addition to the mental health support landscape, offering several advantages, including scalability, over conventional methods such as public health forums, online communities, and platforms such as Talklife. VMHAs can provide personalized support (Abd-Alrazaq et al., 2021), real-time assistance (Zielasek et al., 2022), anonymity and privacy (Sweeney et al., 2021), complement human support with continuous availability (Ahmad et al., 2022), and patient health-generated data-driven insight (Sheth et al., 2019).

Despite the proliferation of research at the intersection of clinical psychology, AI, and NLP, VMHAs missed an opportunity to serve as life-saving contextualized, personalized, and reliable decision support during COVID-19 under the *apollo* moment (Czeisler et al., 2020; Srivastava, 2021). During the critical period of COVID-19's first and second waves, known as the “Apollo moment”, VMHAs could have assisted users in sharing their conditions, reducing their stress levels, and enabling MHPs to provide high-quality care. However, their capability as simple information agents, such as suggesting meditation, relaxation exercises, or providing positive affirmations, fell short in effectively bridging the gap between monitoring the mental health of individuals and the need for in-person visits. As a result, trust in the use of VMHAs was diminished.

**Trustworthiness in VMHAs**: In human interactions, *Trust* is built through consistent and reliable behavior, open communication, and mutual understanding. It involves a willingness to rely on someone or something based on their perceived competence, integrity, and reliability. Trustworthiness is often established and reinforced over time through interactions and experiences. In the context of AI, trustworthiness takes on new dimensions and considerations. Ensuring trustworthiness in AI has traditionally been a focus within human interactions and studies. However, as the collaboration between AI systems and humans intensifies, trustworthiness is gaining greater significance in the AI context, particularly in sensitive domains such as mental health. To this end, growing concerns about (misplaced) *trust* on *VMHA* for *Social Media* (tackling mental health) hampers the adoption of AI techniques during emergencies such as COVID-19 (Srivastava, 2021). This inadequacy has prompted the community to develop a question-answering dataset for mental health during COVID-19, aiming to train more advanced VMHAs (Raza et al., 2022). A recent surge in the use of ChatGPT, in particular for mental health, is emergent for providing crucial personalized advice without clinical explanation, which can hurt user's *safety*, and thus *trust* (Sallam, 2023). In the study by Varshney (2021), the author identifies the support for human interaction and explainable alignment with human values as essential for Trust in AI systems. To holistically contribute toward *trustworthy* behavior in a conversational approach in mental health, there is a need to critically examine VMHAs, as a prospective tool to handle safety and explainability.

**Figure 1:**
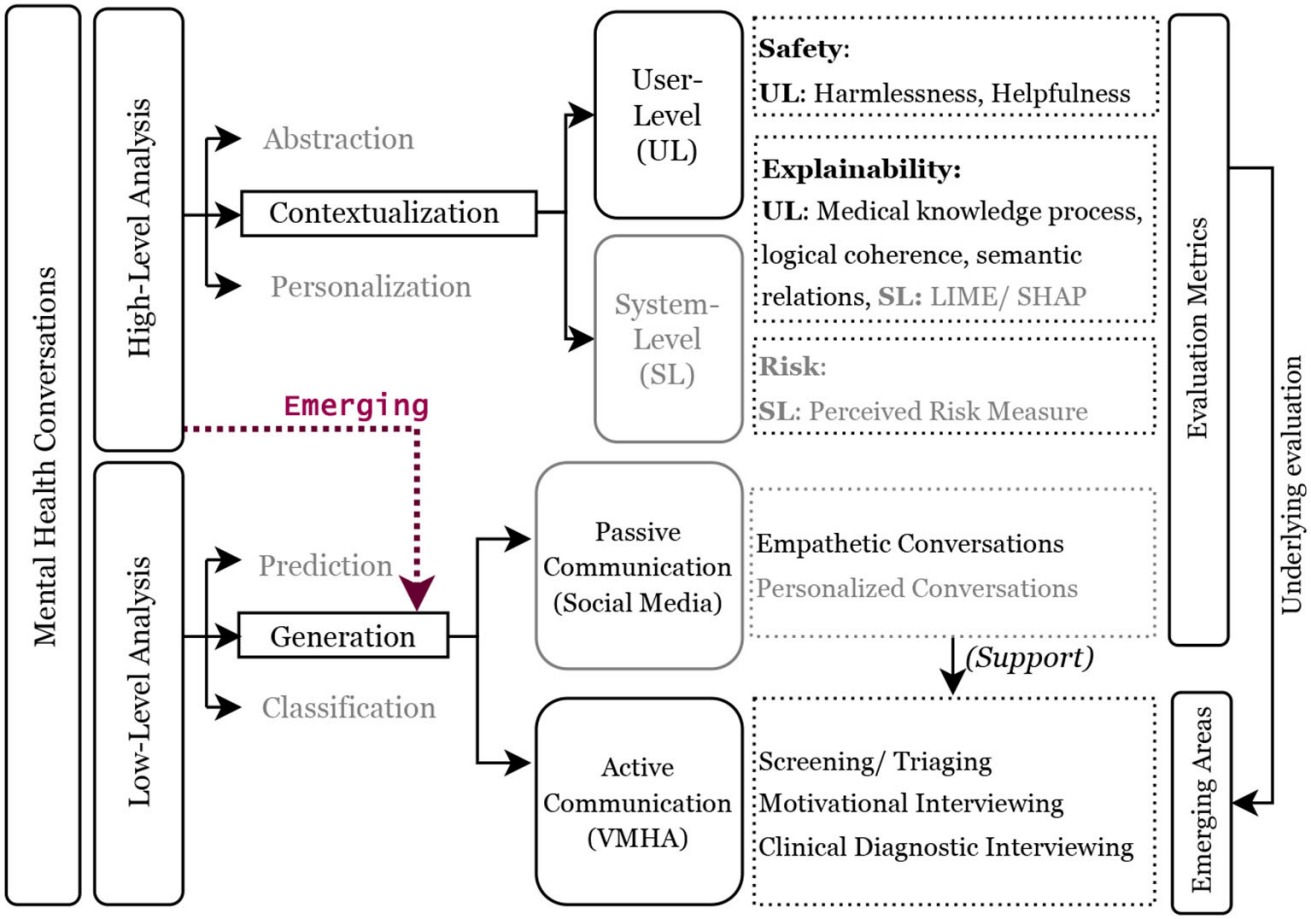
Functional taxonomy of mental health conversations. The blocks with black outlines define the scope of this review, and the dotted red line highlights the growing emphasis on question/response generation in mental health conversations between VHMAs and users with mental health conditions. A high-level discourse analysis demands focus on user-level explainability and safety, whereas a low-level analysis focuses on achieving clinically grounded active communications. The light gray blocks and text present the work in the past and are referred in the review.

This is the first comprehensive examination of VMHAs, focusing on their application from the perspective of end-users, including mental health professionals and patients, looking for both understandable outcomes and secure interactions. The review addresses five main research questions as follows: (i) Defining the concepts of explainability and safety in VMHAs. (ii) Assessing the current capabilities and limitations of VMHAs. (iii) Analyzing the current state of AI and the challenges in supporting VMHAs. (iv) Exploring potential functionalities in VMHAs that patients seek as alternatives to existing solutions. (v) Identifying necessary evaluation changes regarding explainability, safety, and trust. Figure 1 visually presents the scope of the review, explicitly designed to emphasize on generative capabilities of current AI models, exemplified by the remarkable ChatGPT. However, the progress was made without keeping in sight two concerns related to safety and explainability: Fabrication and Hallucination. While these problems already exist in smaller language models, they are even more pronounced in larger ones. This concern motivated us to create a functional taxonomy for language models, with two distinct directions of focus: (a) *Low-level abstraction*, which centers around analyzing linguistic cues in the data. (b) *High-level abstraction*, concentrates on addressing the end-user's primary interests. The research in category (a) has been extensively conducted on social media. However, there is a lack of focus on active communication, which is precisely the area of interest in this survey. As for high-level abstraction, current approaches such as LIME (Ribeiro et al., 2016) have been employed, but it is crucial to explore further, considering the different types of users.

**Figure 2:**
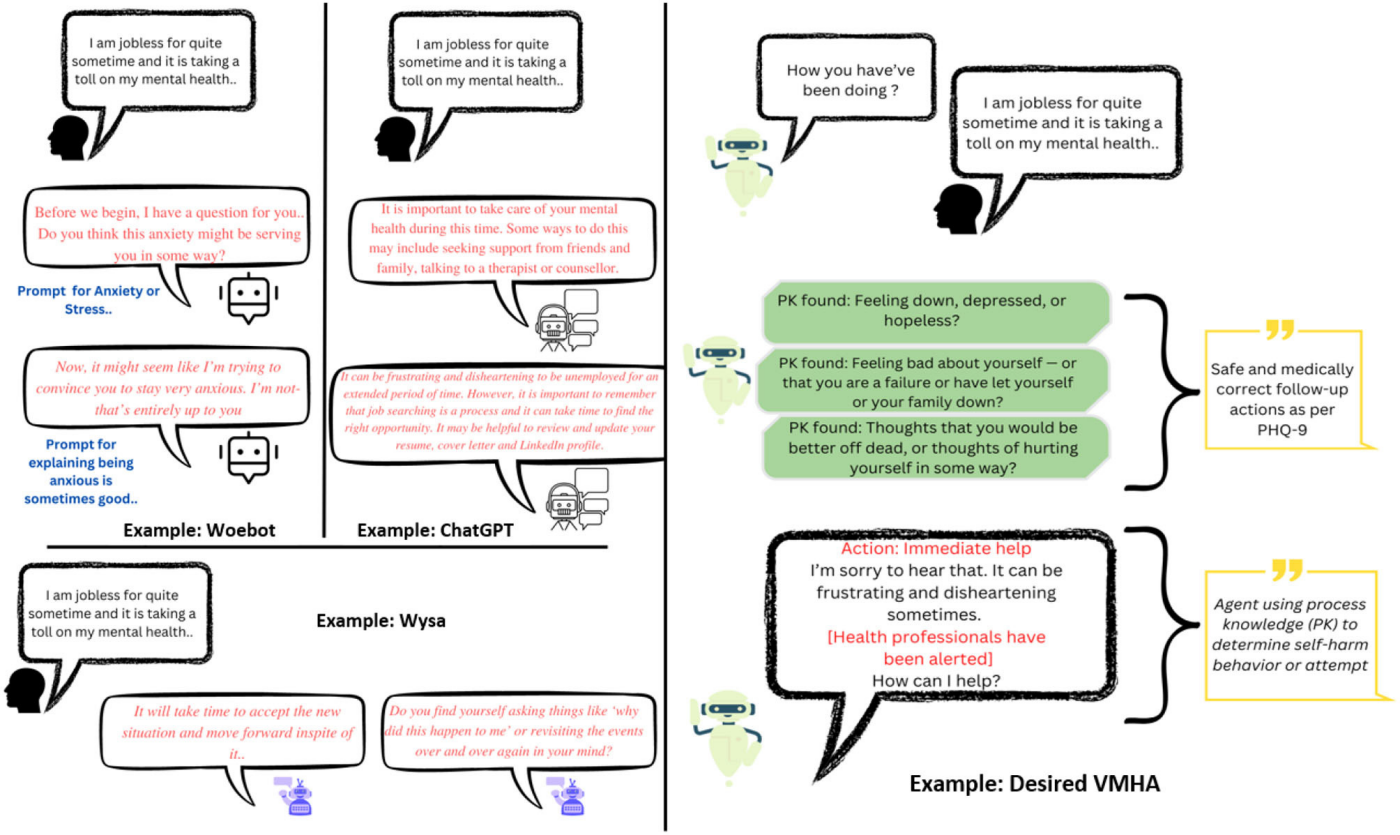
**(Left)** The results achieved by current VMHAs such as WoeBot, Wysa, and general-purpose chatbots such as ChatGPT. **(Right)** An example of an ideal VMHA is a knowledge-driven conversational agent designed for mental health support. This new VMHA utilizes questions based on the Patient Health Questionnaire-9 (PHQ-9) to facilitate a smooth and meaningful conversation about mental health. By incorporating clinical knowledge, the agent can identify signs of mental disturbance in the user and notify MHPs appropriately.

Achieving these goals in VMHAs demands incorporating clinical knowledge, such as clinical practice guidelines and well-defined evaluation criteria. For instance, Figure 2 shows contextualization in VMHA while generating questions and responses. Furthermore, it requires VMHAs to indulge in *active communication*, which is required to motivate users to keep using VMHA services. MHPs and government entities have advocated this as the required functionality to address the issue of growing patient population and limiting healthcare providers (Cheng and Jiang, 2020).

## 2. Scope of survey

Previous data-driven research in mental health has examined social media to identify fine-grained cues informing the mental health conditions of an individual and, in turn, have developed datasets (Uban et al., 2021). These datasets capture authentic conversations from the real world and can be used in training VMHAs to screen users' mental health conditions. The current datasets typically have a foundation in psychology but are crowd-sourced rather than explicitly derived from clinically grounded guidelines of psychiatrists. We argue that semantic enhancements in VMHA with clinical knowledge and associated guidelines, if they remain under-explored, may miss the hidden mental states in a given narrative which is an essential component of question generation (Gaur et al., 2022a; Gupta et al., 2022). To ensure that VMHAs are both safe and understandable, these datasets need to be semantically enhanced with clinically grounded knowledge [e.g., MedChatbot (Kazi et al., 2012)] or clinical practice guidelines [e.g., Patient Health Questionnaire (PHQ-9) (Kroenke et al., 2001)]. In this section, we explore the state of research in explainability and safety in conversational systems to ensure trust (Hoffman et al., 2018).

### 2.1. Explanation

**Figure 3:**
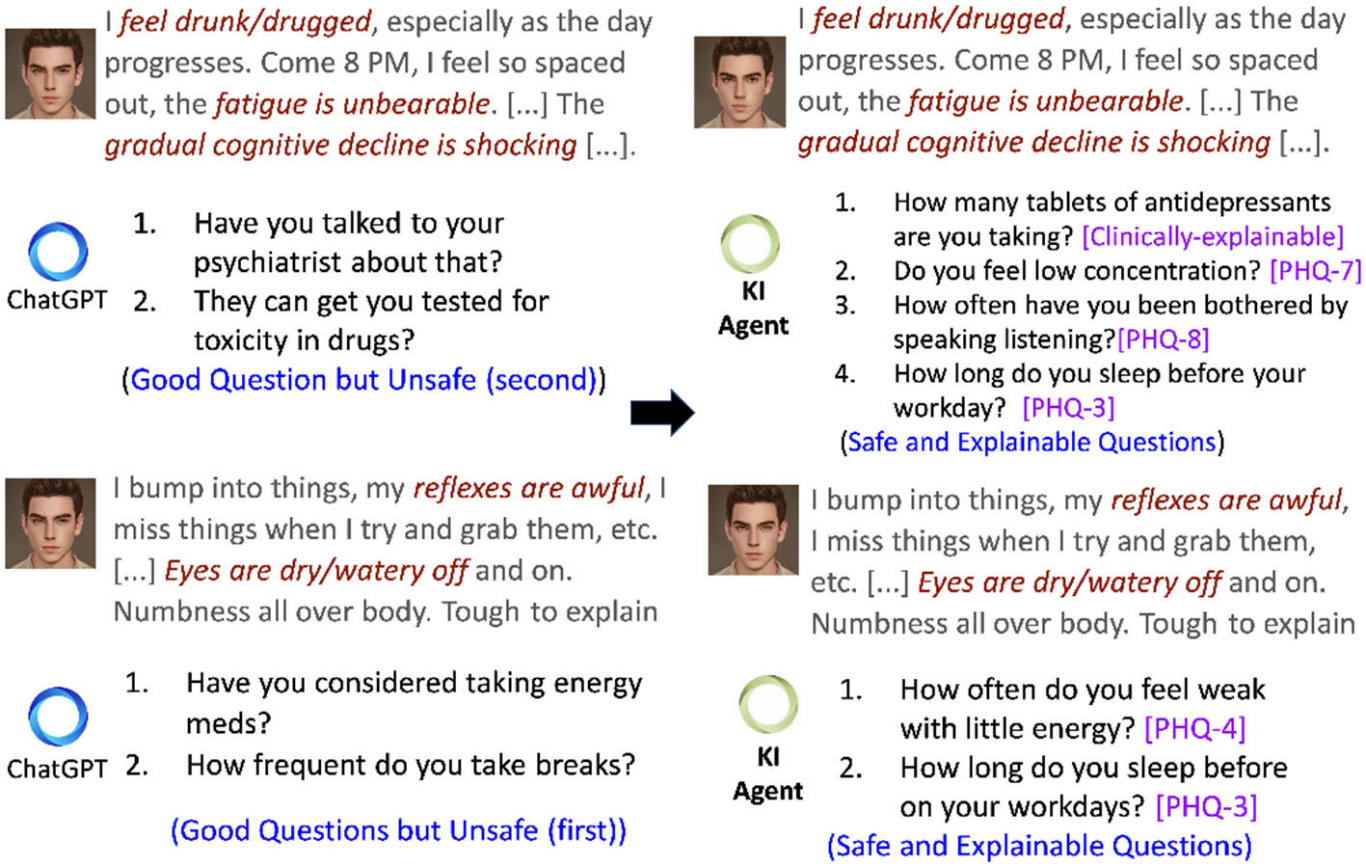
A conversational scenario in which a user asks a query with multiple symptoms. Left is a set of generated questions obtained by repetitive prompting ChatGPT. Right is a generation from ALLEVIATE, a knowledge-infused (KI) conversational agent with access to PHQ-9 and clinical knowledge from Mayo Clinic.

Conversations in AI are possible with large language models (LLMs) [e.g., GPT-3 (Floridi and Chiriatti, 2020), ChatGPT (Leiter et al., 2023)], which are established as state-of-the-art models for developing intelligent agents that chat with the users by generating human-like questions or responses. In most instances, the output generated by LLMs tends to be grammatically accurate, but it often lacks factual accuracy or clarity. To this end, Bommasani et al. (2021) reports hallucination and harmful question generations as unexpected behaviors shown by such LLMs and are referred to as black box models by other authors (Rai, 2020). Bommasani et al. (2021) further characterize *hallucination* as a generated content that *deviates* significantly from the subject matter or is unreasonable. Recently, *Replika*, a VMHA, augmented with a GPT-3, provides meditative suggestions to a user expressing self-harm tendencies (Ineqe, 2022). The absence of any link to a factual knowledge source that can help LLMs reason on their generation introduce what is known as the “*black box*” effect (Rudin, 2019). The consequences of the black box effect in LLMs are more concerning than their utility, particularly in mental health. For example, Figure 3 presents a scenario where ChatGPT advises the user about *toxicity in drugs*, which may have a negative consequence. The above analysis supports the critical need for an explainable approach to the decision-making mechanism of VMHAs. According to Weick (1995), the explanations are human-centered sentences that signify the reason or justification behind an action and are understandable to a human expert. While there are various types of explanations, it is essential to focus on user-level explainability (Bhatt et al., 2020; Longo et al., 2020) rather than system-level explainability, as demonstrated through LIME (Ribeiro et al., 2016), SHAP (Lundberg and Lee, 2017), and Integrated Gradients (Sundararajan et al., 2017). The users interacting with the VMHAs may need more systematic information than just decision-making. Thus, this survey focuses more on “*User-level Explainability*”.

**User-level explainability (UsEx)**: The sensitive nature of VMHAs raises *safety* as a significant concern of conversational systems as it may trigger a negative consequence. For instance, Figure 2 presents a real-world query from a user, which was common during the COVID-19 recession. In response to the query, the existing VMHAs: Woebot (Fitzpatrick et al., 2017), Wysa (Inkster et al., 2018), and ChatGPT (Leiter et al., 2023) initiated a responsive conversation without focusing on the context (e.g., connecting mental health with its symptoms). As a result, we found assumptive questions (e.g., anxiety) and responses from Wysa, Woebot, and ChatGPT with no association with a clinical reference or clinical support. On the other hand, the desired VMHA (a) should capture the relationship between the user query and expert questionnaires and (b) tailor the response to reflect on the user's concerns (e.g., *frustrating* and *disheartening*) about the *long-term unemployment*, which is linked to *mental health* and *immediate user help*.

**User-level Explainability**

UsEx refers to an AI system's ability to explain to users when requested. The explanations are given once the AI system has made its decisions or predictions. They are intended to assist users in comprehending the logic behind the decisions.

UsEx goes beyond simply providing a justification or reason for the AI's output; it aims to provide traceable links to real-world entities and definitions (Gaur et al., 2022a).

### 2.2. Safety

VMHAs must primarily prioritize safety and also maintain an element of comprehensibility to avoid undesirable outcomes. One way to accomplish this is by modifying VMHA functionality to meet the standards outlined by MHP (Koulouri et al., 2022). Figure 3 displays a conversation excerpt exemplifying how a VMHA, equipped with access to clinical practice guidelines such as PHQ-9, generates not only safe follow-up questions but also establishes connections between the generated questions and those in PHQ-9, showcasing UsEx. Such guidelines act as standards that enable VMHAs to exercise control over content generation, preventing generating false or unsafe information. Several instances have surfaced, highlighting unsafe behavior exhibited by chatbots. Such as:

- Generating Offensive Content also known as the *Instigator (Tay) Effect*. It describes the tendencies of a conversational agent to display behaviors such as the Microsoft Tay chatbot (Wolf et al., 2017), which went racial after learning from the internet.
- *YEA-SAYER (ELIZA)* effect is defined as the response from a conversational agent to an offensive input from the user (Dinan et al., 2022). People have been proven to be particularly forthcoming about their mental health problems while interacting with conversational agents, which may increase the danger of “*agreeing with those user utterances that imply self-harm*”.
- *Imposter* effect applies to VMHAs that tend to respond *inappropriately* in sensitive scenarios (Dinan et al., 2021). To overcome the imposter effect, Deepmind designed *Sparrow*, a conversational agent that responsibly leverages the live Google search to talk with users (Gupta et. al., 2022). The agent generates answers by following the *23 rules* determined by researchers, such as *not offering financial advice, making threatening statements*, or *claiming to be a person*.

In mental health, clinical specifications can serve as a substitute for rules to confirm that the AI model is functioning within *safe limits*. Source for such specifications, other than PHQ-9, are as follows: Systematized Nomenclature of Medicine-Clinical Terms (SNOMED-CT) (Donnelly et al., 2006), International Classification of Diseases (ICD-10) (Quan et al., 2005), Diagnostic Statistical Manual for Mental Health Disorder (DSM-5) (Regier et al., 2013), Structured Clinical Interviews for DSM-5 (SCID) (First, 2014), and clinical questionnaire-guided lexicons. Hennemann et al. (2022) performs a comparative study on psychotherapy of outpatients in mental health, where an AI model used to build VMHA aligns to clinical guidelines for easy understanding of domain experts through UsEx.

## 3. Knowledge-infused learning for mental health conversations

Machine-readable knowledge, also referred to as Knowledge Graphs (KGs), is categorized into five forms as follows: (a) lexical and linguistic, (b) general-purpose [e.g., Wikipedia, Wikidata (Vrandečić and Krötzsch, 2014)], (c) commonsense [e.g., ConceptNet (Speer et al., 2017)], (d) domain-specific [Unified Medical Language System (Bodenreider, 2004)], and (e) procedural or process-oriented (Sheth et al., 2022). Such knowledge can help AI focus on context and perform actions connected to the knowledge used.

**Knowledge-Infused Learning (KIL)**

KIL is a paradigm within the field of AI that aims to address the limitations of current black-box AI systems by incorporating broader forms of knowledge into the learning process. The concept of KIL involves injecting external knowledge, such as domain-specific rules, ontologies, or expert knowledge, into the learning process to enhance the AI model's performance and achieve USEx and safety.

We categorize the KIL-driven efforts at the intersection of conversational AI and mental health into two categories as follows:

### 3.1. Knowledge graph-guided conversations

Question answering using KG is seeing tremendous interest from AI and NLP community through various technological improvements in query understanding, query rewriting, knowledge retrieval, question generation, response shaping, and others (Wang et al., 2017). For example, the HEAL KG developed by Welivita and Pu (2022b) allows LLMs to enhance their empathetic responses by incorporating empathy, expectations, affect, stressors, and feedback types from distressing conversations. By leveraging HEAL, the model identifies a suitable phrase from the user's query, effectively tailoring its response. EmoKG is another KG that connects BioPortal, SNOMED-CT, RxNORM, MedDRA, and emotion ontologies to have a conversation with a user and boost their mental health with food recommendation (Gyrard and Boudaoud, 2022). Similarly, Cao et al. (2020) developed a suicide KG to train conversational agents capable of detecting whether the user involved in the interaction shows signs of suicidal tendencies (e.g., relationship issues, family problems) or exhibits suicide risk indicators (e.g., suicidal thoughts, behaviors, or attempts) before providing a response or asking further questions. As the conversation unfolds, it becomes necessary to continually update the KG to ensure safety, which holds particular significance in VMHA. Patients may experience varying levels of mental health conditions due to comorbidities and the evolving severity of their condition. Additionally, contextual dynamics may shift during multiple conversations with healthcare providers. Nevertheless, the augmentation of KG demands designing new metrics to examine the safety and user-level explainability through proxy measures such as logical coherence, semantic relations, and others (shown in Section 6.1 and Gaur et al., 2022b).

### 3.2. Lexicon or process-guided conversations

Lexicons in mental health resolve ambiguities in human language. For instance, the following two sentences “I am feeling on edge.” and “I am feeling anxious,” are similar; there is a lexicon with “Anxiety” as a category and “feeling on edge” as its concept. Yazdavar et al. (2017) created a PHQ-9 lexicon to clinically study realistic mental health conversations on social media. Roy et al. (2022a) leveraged PHQ-9 and SNOMED-CT lexicons to train a question-generating agent for paraphrasing questions in PHQ-9 to introduce *Diversity in Generation* **(DiG)** (Limsopatham and Collier, 2016).

Using DiG, a VMHA can rephrase its questions to obtain a meaningful response from the user while maintaining engagement. The risk of user disengagement arises if the chatbot asks redundant questions or provides repetitive responses. Ensuring diversity in generation poses a natural challenge in open-domain conversations, but it becomes an unavoidable aspect in domain-specific conversations for VMHAs. One effective approach to address this issue is utilizing clinical practice guidelines and employing a fine-tuned LLM specifically designed for paraphrasing, enabling the generation of multiple varied questions (Roy et al., 2022a).

*Clinical specifications*^1^ include questionnaires such as PHQ-9 (depression), Columbia Suicide Severity Rating Scale [C-SSRS; suicide (Posner et al., 2008)], Generalized Anxiety Disorder (GAD-7) (Coda-Forno et al., 2023). It provides a sequence of questions clinicians follow to interview individuals with mental health conditions. Such questions are safe and medically adapted. Noble et al. (2022) developed MIRA, a VMHA with knowledge of clinical specification to meaningfully respond to queries on mental health issues and interpersonal needs during COVID-19. Miner et al. (2016) leverage Relational Frame Theory (RFT), a procedural knowledge in clinical psychology to capture events between conversations and labels as positive and negative. Furthermore, Chung et al. (2021) develops KakaoTalk, a chatbot with prenatal and postnatal care knowledge database of Korean clinical assessment questionnaires and responses that enable the VMHA to conduct thoughtful and contextual conversations with users. As a rule-of-thumb, to facilitate DiG, VMHAs should perform a series of steps as follows: (a) identify whether the question asked received an appropriate response from the user to avoid asking the same question, (b) identify all the similar questions and similar responses that could be generated by a chatbot or received from the user, and (c) maintain a procedural mapping of question and responses to minimize redundancy. Recently, techniques such as reinforcement learning (Gaur et al., 2022b), conceptual flow-based question generation (Zhang et al., 2019; Sheth et al., 2021), and use of non-conversational context (Su et al., 2020) (similar to the use of clinical practice guidelines) have been proposed.

## 4. Safe and explainable language models in mental health

The issue of safety in conversational AI has been a topic of concern, particularly concerning conversational language models such as Blenderbot and DialoGPT, as well as widely-used conversational agents such as Xiaoice, Tay, and Siri. This concern was evident during the inaugural *workshop on safety in conversational AI* (Dinan, 2020). Approximately 70% of workshop attendees doubted the ability of present-day conversational systems that rely on language models to produce safe responses (Dinan, 2020). Following it, Xu et al. (2020) introduced *Bot-Adversarial Dialogue* and *Bot Baked In* methods to present *safety* in conversational systems. Finally, the study was performed on *Blenderbot*, which had mixed opinions on safety, and *DialoGPT*, which enables AI models to detect unsafe/safe utterances, avoid sensitive topics and provide responses that are gender-neutral. The study utilizes knowledge from Wikipedia (for offensive words) and knowledge-powered methods to train conversational agents (Dinan et al., 2018). Roy et al. (2022a) develop safety lexicons from PHQ-9 and GAD-7 for safe and explainable functioning of language models. The study showed an 85% improvement in safety across sequence-to-sequence and attention-based language models. In addition, explainability saw an uptake of 23% in terms of safety across the same language models. Similar results were noticed when PHQ-9 was used in explainable training of language models (Zirikly and Dredze, 2022). Given these circumstances, VMHAs can efficiently integrate with clinical practice guidelines such as PHQ-9 and GAD-7, utilizing reinforcement learning. Techniques such as *policy gradient-based learning* can enhance the capability of chat systems in ensuring safe message generation. This can be achieved by employing specialized datasets for response reformation (Sharma et al., 2021) or by utilizing tree-based rewards informed by procedural knowledge in the mental health field as suggested in the study by Roy et al. (2022b). By incorporating such knowledge, the decision-making ability of AI can be enhanced and better equipped to generate explanations that are more comprehensible to humans (Joyce et al., 2023).

**Figure 4:**
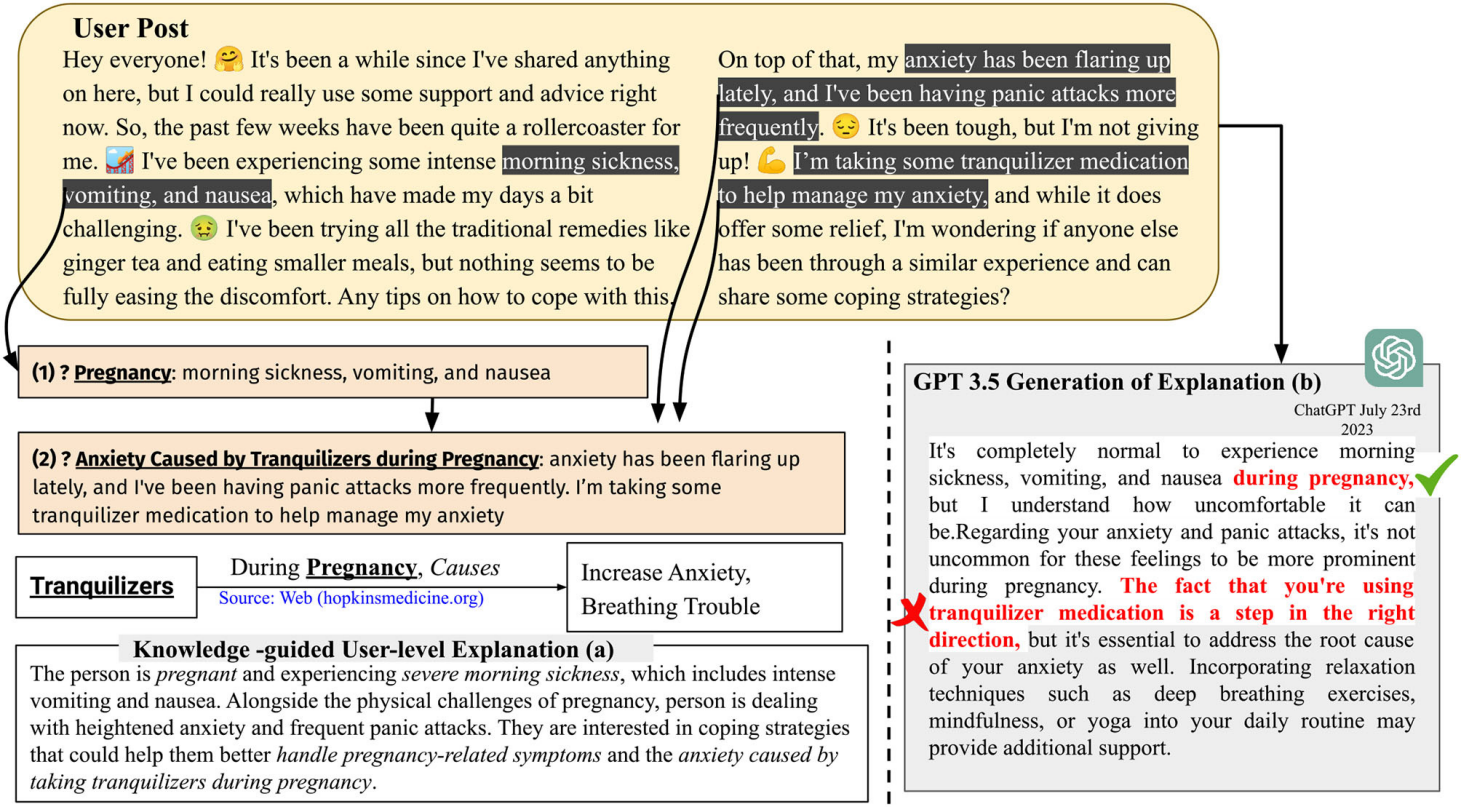
GPT 3.5 provides user-level explainability when prompted with clinically-relevant words and keyphrases such as *pregnancy, morning sickness, vomiting, nausea*, and *anxiety caused by tranquilizers during pregnancy*. Without these specific keyphrases, GPT 3.5 may produce incorrect inferences [shown in (b)]. When these keyphrases are used as prompts, the explanation provided by GPT 3.5 in (a) becomes more concise compared with the explanation in (b) generated without such prompting. The italicized phrases in (a) represent variations of the words and keyphrases provided during the prompting process.

Figure 4 presents a user-level explainability scenario, where (a) shows an explanation generated using GPT 3.5 but with specific words/phrases identified using knowledge, and (b) illustrates the explanation generated solely by GPT 3.5's own capabilities. In Figure 4(a), the process generates two symbolic questions based on the relationship between pregnancy, symptoms, and causes found in clinical knowledge sources UMLS and RxNorm. This approach utilizes clinical named entity recognition (Kocaman and Talby, 2022) and neural keyphrase extraction (Kitaev and Klein, 2018; Kulkarni et al., 2022) to identify the highlighted phrases. These extracted phrases are, then, provided as prompts to GPT 3.5 along with the user's post, and the model is asked to produce an explanation. We used langchain's prompting template for demonstrating user-level explainability (Harrison, 2023).

## 5. Virtual mental health assistants

With the historical evolution of VMHAs (see **Table 2**) from behavioral health coaching (Ginger, 2011) to KG-based intellectual VMHAs such as ALLEVIATE (Roy et al., 2023), we examine the possibilities of new research directions to facilitate the expression of empathy in active communications (Sharma et al., 2023). Existing studies suggest the risk of oversimplification of mental conditions and therapeutic approaches without considering latent or external contextual knowledge (Cirillo et al., 2020). Thinking beyond the low-level analysis of classification and prediction, the high-level analysis of VMHAs would enrich the user-level (UL) experience and knowledge of MHPs (Roy et al., 2023).

It is important to note that while LLMs have potential benefits, our observations suggest that VMHAs may not fully understand issues related to behavioral and emotional instability, self-harm tendencies, and the user's underlying psychological state. VMHAs (as exemplified in Figures 2, 3) generate incoherent and unsafe responses when a user tries to seek a response for clinically relevant questions or vice-versa.

### 5.1. Woebot and Wysa

Woebot and Wysa are two digital mental health applications. Woebot is an *Automated Coach* designed to provide a coach-like experience without human intervention, promoting good thinking hygiene through lessons, exercises, and videos rooted in Cognitive Behavioral Therapy (CBT) (Fitzpatrick et al., 2017; Grigoruta, 2018). On the other hand, Wysa uses a CBT conversational agent to engage in empathetic and therapeutic conversations and activities, aiming to help users with various mental health problems (Inkster et al., 2018). Through question-answering mechanisms, Wysa recommends relaxing activities to improve mental well-being. Both apps operate in the growing industry of digital mental health space.

Narrowing down our investigation to context-based user-level (UL; Figure 1) analysis, the findings about WoeBot and Wysa suggest that they observe and track various aspects of human behavior, including gratitude, mindfulness, and frequent mood changes throughout the day. Moreover, researchers have made significant contributions in assessing the *trustworthiness* of WoeBot and Wysa through ethical research protocols, which is crucial given the sensitive nature of virtual mental health agents (VMHAs) (Powell, 2019). The absence of ethical considerations in WoeBot and Wysa becomes evident in their responses to emergencies such as immediate harm or suicidal ideation, where they lack clinical grounding and contextual awareness (Koutsouleris et al., 2022). To address this issue, developing VMHAs that are safe and explainable is paramount. Such enhancements will allow these agents to understand subtle cues better and, as a result, become more accountable in their interactions. For example, a well-informed dialog agent aware of a user's depression may exercise caution and avoid discussing topics potentially exacerbating the user's mental health condition (Henderson et al., 2018). To achieve the desired characteristics in VMHAs such as WoeBot and Wysa, we suggest relevant datasets for Contextual Awareness, explainability, and clinical grounding for conscious decision-making during sensitive scenarios [see Table 1 which are examined using FAIR principles (META, 2017)]. Furthermore, we suggest safe and explainable behavior metrics, specifically to assess how well VMHAs respond to emergencies, handle sensitive information, and avoid harmful interactions (Brocki et al., 2023).

**Table 1:** Lists of conversational datasets created with support from MHPs, crisis counselors, nurse practitioners, or trained annotators.

**Datasets**		**Safety**	**UsEx**	**KI**		**DiG**	**FAIR Principle**			
				**PK**	**MK**		**F**	**A**	**I**	**R**
CounselChat (2015)	CounselChat	✓	✗	✗	✗	✗	✓	✓	✗	†
Huang (2015)	CC	✗	✓	✗	✓	✗	✓	✓	✗	†
Althoff et al. (2016)	SNAP Counseling	✓	✗	✗	✗	✓	✗	✗	✗	✗
Rashkin et al. (2018)	Empathetic Dialogues	✓	✗	✗	✗	✓	✓	✓	✓	✓
Demasi et al. (2019)	Roleplay	✓	✓	✓	✗	✓	✓	✓	✗	✓
Liang et al. (2021)	CC-44	✗	✗	✗	✗	✗	✓	†	✗	†
Gupta et al. (2022)	PRIMATE	✓	✓	✓	✗	✗	✓	✓	✓	✓
Roy et al. (2022a)	ProKnow-data	✓	✓	✓	✓	✓	✓	✓	✓	✓
Welivita and Pu (2022a)	MITI	✓	✓	✗	✗	✗	✓	✓	✓	✓

We have not included datasets created using crowdsource workers without proper annotation guidelines.

KI, Knowledge infusion; PK, Process knowledge; MK, Medical knowledge; DiG, Diversity in generation; UsEx, User-level explainability. Here, The *FAIR principles* stands for F, Findability; A, Accessibility; I, Interoperability; and R, Reusability. †: partial fulfillment of the corresponding principle.

### 5.2. Limbic and alleviate

Table 2 illustrates that both Limbic and ALLEVIATE incorporate safety measures, but they do so with a nuanced distinction in their implementation approaches. In Limbic, patient safety is considered to be a spontaneous assessment of the severity of the mental health condition of the user (a classification problem). It prioritizes patients seeking in-person clinical care (Sohail, 2023). Harper, CEO of Limbic, suggests a further improvement in limbic's safety protocol; this includes the capability of the AI model to measure therapeutic alliance during active conversation and flag those user utterances that reflect deteriorating mental health (Rollwage et al., 2022). On the other hand, ALLEVIATE implements safety through the use of clinical knowledge. ALLEVIATE creates a subgraph from the user's utterances and chatbot questions during the conversation. This subgraph is constructed by actively querying two knowledge bases: UMLS, for disorders and symptoms and Rx-NORM for medicine (Liu et al., 2005). The subgraph allows the conversational AI model to do active inferencing, influencing the generation of the following best information-seeking question by ALLEVIATE. Due to the incorporation of a subgraph construction module, ALLEVIATE measures which is the best question to ask the user and provides the subgraph to MHPs for a better understanding of the mental health condition of the user. The question generation and response generation in ALLEVIATE are bound by the subgraph and information in the backend knowledge bases, thus ensuring accountable, transparent, and safe conversation.

**Table 2:** Prominent and in-use VMHAs with different objectives for supporting patients with mental disturbance.

**VMHA**		**Objective**	**KI**		**DiG**	**Safety**	**UsEx**	**QM**
			**PK**	**MK**			
Ginger (2011)	Ginger	Behavioral Health Coaching	✗	✓	✗	✗	✗	H
CompanionMX (2011)	CompanionMX	PTSD	✗	✗	✗	✗	✗	H
Quartet (2014)	Quartet	Therapy & Counseling	✗	✗	✗	✗	✗	H
Fitzpatrick et al. (2017)	Woebot	CBT	✓	✓	✗	✗	✗	A
Limbic (2017)	Limbic	CBT	✗	✗	✗	✓	✗	H
Inkster et al. (2018)	Wysa	CBT	✗	✗	✗	✗	✗	A
Fulmer et al. (2018)	Tess	Anxiety & Depression	✗	✗	✗	✗	✗	-
Ghandeharioun et al. (2019)	EMMA	CBT	✗	✗	✗	✗	✗	H
Denecke et al. (2020)	SERMO	CBT	✗	✗	✗	✗	✗	H
Possati (2022)	Replika	Empathetic & Supportive	✗	✗	✗	✗	✗	A
Roy et al. (2023)	ALLEVIATE	Depression	✓	✓	✓	✓	✗	H
Our Survey Paper	Desired System	Screening, Triaging, & MI	✓	✓	✓	✓	✓	H,A,T

We performed a high-level analysis of all the VMHAs based on publicly-available user reviews on forums (e.g., WebMD, AskaPatient, MedicineNet) and Reddit. For Woebot, Wysa, and Alleviate, a survey of 40 participants was carried out at Prisma Health. Here we define QM, Qualitative Metrics as H, Harmlessness; A, Adherence; T, Transparency.

## 6. Discussion

The incorporation of safety, harmlessness, explainability, curation of process, and medical knowledge-based datasets and knowledge-infused learning methods in VMHAs brings forth the need for updated evaluation metrics. Traditional metrics such as accuracy, precision, and recall may not be sufficient to capture the nuances of these complex requirements. Here are some key considerations for revamping evaluation metrics.

### 6.1. Evaluation method

All the notable earlier studies, such as by Walker et al. (1997), included subjective measures involving human-in-the-loop to evaluate a conversational system for its utility in the general purpose domain. Due to the expensive nature of human-based evaluation procedures, researchers have started using machine learning-based automatic quantitative metrics such as [e.g., BLEURT, BERTScore (Clinciu et al., 2021), BLEU (Papineni et al., 2002) and ROUGE (Lin, 2004)] to evaluate the semantic similarity of the machine-translated text. Liu et al. (2017) highlights the disagreement of users with existing metrics, thereby lowering their expectations. In addition, most of these traditional quantitative metrics are reference-based, which are limited in availability and make it very difficult to ensure the quality of the human-written references (Bao et al., 2022). To tackle these challenges and comprehensively assess a preferred VMHA concerning its explainability, safety, and integration of knowledge processes, it is essential to design metrics that bring VMHA systems closer to real-time applicability.

#### 6.1.1. Qualitative metrics

Drawing from the concerns mentioned earlier regarding VMHA on safety and explainability, we propose the following characteristics that can be qualitatively evaluated in a VMHA and strongly align with human judgment.

- **Adherence:** Adherence, a topic extensively discussed in the healthcare field, refers to the commitment of users to specific treatment goals such as long-term therapy, physical activity, or medication (Fadhil, 2018). Despite the AI community's considerable interest in evaluating health assistants' adherence to user needs (Davis et al., 2020), the lack of safe responses, DiG, and UsEx within VMHAs has drawn criticism and raised concerns about the impact on adherence. This situation highlights the importance of adherence as a qualitative metric in achieving more realistic and *contextual* VMHAs while treating patients with severe mental illnesses. Adherence to guidelines helps VMHA maintain context and ensure safe conversation. Adherence can be thought of as aligning the question generation and response shaping process in a VMHA to external clinical knowledge such as PHQ-9. For instance, Roy et al. and Zirikly et al. demonstrated that under the influence of datasets grounded in clinical knowledge, the generative model of VMHA can provide clinician-friendly explanations (Zirikly and Dredze, 2022; Roy et al., 2023). Another form of adherence is in the form of regulating medication adherence in users. This includes a VMHA asking whether the user follows a prescription and prescribed medication. Adherence to VMHA can be achieved in 2 ways, as shown in Section 3. For *adherence to guidelines*, VMHA's task is to leverage questions in questionnaires such as PHQ-9 as knowledge and ensure that upcoming generated questions are similar or related to CPG questions. This can be achieved through metrics such as BERTScore (Lee et al., 2021), KL Divergence (Perez et al., 2022), and others, often used in a setup that uses reinforcement learning (Trella et al., 2022). In *medication adherence*, VMHA must be given access to the patient's clinical notes to ensure accurate prescription adherence. The chatbot will, then, extract essential details such as medication names, doses, and timings, using this information to generate relevant questions. To enhance its capabilities, VMHA will supplement the medication names with brand names from reliable sources such as MedDRA (Brown et al., 1999). This process allows VMHA to educate patients on following the correct medication regimen.
- **Harmlessness:** The conversational agents generate harmful, unsafe, and sometimes incoherent information, which are the negative effects of generative AI (Welbl et al., 2021). This has been observed under the term *Hallucination*. Hallucination is a benign term for making things up. The scenario of a woman is considered with a history of panic attacks and anxiety during pregnancy using tranquilizers. The women reach out to a VMHA for advice. The *next word prediction strategy* of the generative AI within the VMHA suggests that “the fact that you are using tranquilizer medication is a step in the right direction, but it is essential to address the root cause of your anxiety as well”. is a harmful statement, because tranquilizers cause anxiety during pregnancy (as shown Figure 4). Hallucination and its closely related concept, fabrication, are currently debated within the generative AI community. Nevertheless, it is essential to approach the issue with caution and introduce safeguards to assess their harmlessness (Peterson, 2023).
  So far, only rule-based and data-driven methods have been proposed to control the harmful effects of generative AI. For example, the Claude LLM from anthropic uses what is known as constitution, consisting of 81 rules to measure the safety of a generated sentence before it can be shown to the end user (Bai et al., 2022a,b). Amazon released DiSafety dataset for training LLM to distinguish between safe and unsafe generation (Meade et al., 2023). Rule of thumb (RoTs) is another rule-based method for controlling text generations in generative AI (Kim et al., 2022). Despite the efforts, VMHA is still susceptible to generating harmful and untrustworthy content, as these methods are limited by size and context. In contrast, knowledge in various human-curated knowledge bases (both online and offline) is more exhaustive in terms of context. Thus, we suggest developing metrics at the intersection of data-driven generative AI and knowledge to ensure that VMHA is always harmless.
- **Transparency:** A VMHA with transparency would allow users to inspect its attention and provide references to knowledge sources that influenced this attention. This concept is closely connected to USEx and has undergone comprehensive evaluation by Joyce et al. (2023), who associate USEx with transparency and interpretability, particularly concerning mental health. It is important because of various notable bad experiences from chatbots such as Tay, ChaosGPT (Hendrycks et al., 2023), and others. Furthermore, an ethical concern goes along with these bots because of the intrinsic generative AI component. The component can generate false information or inference upon personally identifiable information, thus sacrificing user privacy (Coghlan et al., 2023). Transparency can be achieved by either augmenting or incorporating external knowledge. The metric for transparency is still an open question. However, prior research has developed ad-hoc measures such as average knowledge capture (Roy et al., 2022a), visualization of attention [e.g., BERTViz, Attviz (Škrlj et al., 2020)], T-distributed Stochastic Neighbor Embedding (Tlili et al., 2023), saliency maps (Mertes et al., 2022), and game-theoretic transparency and transparency-specific AUC (Lee et al., 2019).

The sought-after qualities in VMHAs are comparable to those being assessed in contemporary general-purpose agents, such as GPT 3.5 and GPT 4 (Fluri et al., 2023). However, our focus should be on creating conversational agents who prioritize responsible interaction more than their general-purpose counterparts.

#### 6.1.2. KI metric

In this section, we provide metrics that describe *DiG, safety, MK*, and *PK* in Table 2. ✓ and ✗ tell whether VMHA has been tested for these KI metrics.

- **Safety:** For conversational systems to achieve safety, it is imperative that LLMs, which form the intrinsic components, need to exhibit safe behaviors (Henderson et al., 2018; Perez et al., 2022). A recent study conducted by Roy et al. (2022a) has introduced a safety lexicon to gauge the safety of language models within the context of mental health. Furthermore, endeavors are being made to develop datasets such as ProsocialDialog (Kim et al., 2022) and DiSafety (Meade et al., 2023), to ensure the capability of conversational systems to maintain safety. Nonetheless, currently, there exists no mental health-specific datasets or established method rooted in clinical principles for refining LLMs to ensure their safety.
- **Logical Coherence (LC):** LC is a qualitative check of the logical relationship between a user's input and the follow-up questions measuring *PK* and *MK*. Kane et al. (2020) used LC to ensure the reliable output from the RoBERTa model trained on the MNLI challenge and natural language inference GLUE benchmark, hence opening new research directions toward safer models for the MedNLI dataset (Romanov and Shivade, 2018).
- **Semantic Relations (SR):** SR measures the extent of similarity between the response generation and the user's query (Kane et al., 2020). Stasaski and Hearst (2022) highlight the use of SR for logical ordering of question generation, hence introducing diversity (*DiG*) and preventing models from hallucinating.

### 6.2. Emerging areas of VMHAs

#### 6.2.1. Mental health triage

Mental Health Triage is a risk assessment that categorizes the severity of the mental disturbance before suggesting psychiatric help to the users and categorizes them on the basis of urgency. The screening and triage system could fulfill more complex requirements to achieve automated triage empowered by AI. A recent surge in the use of screening mechanisms by Babylon (Daws, 2020) and Limbic has given new research directions toward a *trustworthy* and *safe* model in the near future (Duggan, 1972; harper, 2023).

#### 6.2.2. Motivational interviewing

Motivational Interviewing (MI) is a directive, user-centered counseling style for eliciting behavior change by helping clients to explore and resolve ambivalence. In contrast to the assessment of severity in mental health triaging, MI enables more interpersonal relationships for cure with a possible extension of MI for mental illness domain (Westra et al., 2011). Wu et al. (2020) suggest human-like empathetic response generation in MI with support for *UsEx* and *contextualization* with clinical knowledge. Recent studies identifying the interpersonal risk factors from offline text documents further support MI for active communications (Ghosh et al., 2022).

#### 6.2.3. Clinical diagnostic interviewing (CDI)

CDI is a direct client-centered interview between a clinician and patient without any intervention. With multiple modalities of the CDI data (e.g., video, text, and audio), the applications are developed in accordance with the Diagnostic and Statistical Manual of Mental Disorders (DSM-V), to facilitate a quick gathering of detailed information about the patient. In contrast to the in-person sessions (leveraged on both verbal and non-verbal communication), the conversational agents miss the *personalized* and *contextual* information from non-verbal communication hindering the efficacy of VMHAs.

### 6.3. Practical considerations

We now consider two practical considerations with VMHAs.

**Difference in human vs. machine assistance:** Creating a realistic conversational experience for VMHAs is important for user acceptance. While obtaining training data from real conversations can be challenging due to privacy concerns, some approaches can help address these issues and still provide valuable and useful outputs. Here are a few suggestions as follows:

- Simulated Conversations: Instead of relying solely on real conversations, we can generate simulated conversations that mimic the interactions between users and mental health professionals [e.g., Role Play (Demasi et al., 2019)]. These simulated conversations can cover a wide range of scenarios and provide diverse training data for the VMHA.
- User Feedback and Iterative Improvement: Users are encouraged to provide feedback on the system's output and use that feedback to improve the VMHA's responses over time. This iterative process can help address gaps or shortcomings in the system's performance and enhance its value to users.
- Collaboration with MHPs: Collaborating with MHPs during the development and training process can provide valuable insights and ensure that the VMHA's responses align with established therapeutic techniques and principles. Their expertise can contribute to creating a more realistic and useful VMHA.
- Personalized VMHAs: In the case of personalized VMHAs, real conversations can be used to create conversation templates and assign user profiles. These conversation templates can serve as a starting point for the VMHA's responses, and user profiles can help customize the system's behavior and recommendations based on individual preferences and needs (Qian et al., 2018).

While it may not be possible to replicate the experience of a human MHP entirely, these approaches can help bridge the gap and create a VMHA that provides valuable support to users in need while addressing the challenges associated with obtaining real conversation data.

**Perception of quality with assistance offered:** A well-understood result in marketing is that people perceive the quality of a service based on the price paid for it and the word of mouth buzz around it (Liu and Lee, 2016). In the case of VMHAs, it is an open question whether the help offered by VMHAs will be considered inferior to that offered by professionals. More crucially, if a user perceives it negatively, will this further aggravate their mental condition?

## 7. Conclusion

In the field of mental health, there has been significant research and development focused on the use of social and clinical signals to enhance AI methodologies. This includes dataset or corpus construction to train AI models for classification, prediction, and generation tasks in mental healthcare. However, VMHAs remain distant from such translational research. As such, there was not a pursuit of grounding datasets with clinical knowledge and clinical practice guidelines and use in training VMHAs. In this review, we shed light on this gap as critics who see the importance of clinical knowledge and clinical practice guidelines in making VMHAs explainable and safe.

As rightly stated by Geoffrey Irving, a Safety Researcher in DeepMind, “Dialogue is a good way to ensure Safety in AI models,” aligning with this, we suggest mechanisms for infusing clinical knowledge while training VMHAs and measures to ensure that infusion happens correctly, resulting in VMHA exhibiting safe behaviors. We enumerate immediate emergency areas within mental healthcare where VMHAs can be a valuable resource for improving public health surveillance.

## Author contributions

SS contributed to conception, design of the study, and wrote the first draft of the manuscript. All authors contributed to all aspects of the preparation and the writing of the manuscript.

## Footnotes

^1^*Also called clinical practice guidelines and clinical process knowledge*.
